# Future Model for Efficient and Advanced Space Transportation: Exploration and Perspectives

**DOI:** 10.34133/research.1404

**Published:** 2026-09-02

**Authors:** Haipeng Chen, Xiaowei Wang, Zhengyu Song, Dong Li, Weimin Bao

**Affiliations:** ^1^ China Academy of Launch Vehicle Technology, Beijing, China.; ^2^ China Association of Science and Technology, Beijing, China.

## Abstract

Current space transportation employs a multistage rocket system to deliver the payload to its destination in a single launch, and this integrated model significantly constrains payload capacity for medium-high earth orbits and deep-space missions. However, the medium-high earth orbits and deep-space domains are anticipated to become the next exploration and utilization frontier, which needs large payload capacity and low-cost transportation as support. This study proposes a decoupled design for space access and in-space transfer in a medium-high earth orbit or a deep-space mission. This design introduces a novel in-space transfer transportation system enabled by cryogenic propellant refueling in orbit. In the proposed model, Earth-to-orbit vehicles are responsible for space access, while in-space transfer vehicles handle orbital transfer. This separation allows for independent optimization of vehicle designs according to their specific flight environments. Additionally, on-orbit refueling enables long-term, reusable operations for in-space transfer vehicles. The advantages of the new model are analyzed, demonstrating significant improvements in payload capacity for medium and high earth orbits, as well as substantial reducing transportation costs, holding great practical significance. Relevant design challenges associated are also given, providing a reference for subsequent in-depth researches.

## Introduction

With the rapid advancement of human society and aerospace technologies, humanity’s reliance on outer space is increasing at an unprecedented pace. The demand for space access and development is expanding rapidly, signifying the dawn of a new era characterized by large-scale cislunar exploration and development, as well as extensive deep-space exploration [1–5].

Space transportation capabilities form the cornerstone of all space activities [1–7]. The pursuit of enhanced transportation capacity and reduced costs remains unceasing. Nations worldwide are heavily investing in the development of advanced heavy-lift launch vehicles. The advent of reusable heavy-lift launch vehicles has opened the door for large-scale utilization of low-earth orbit, exemplified by mega-constellation deployment, resulting in intensified global competition [8–10]. Concurrently, medium-high earth orbits and deep-space domains are anticipated to become the next frontier of competition [5,11–15], which also needs large payload capacity and low-cost transportation as support.

Space transportation missions, particularly for medium-high earth orbits and deep-space endeavors, primarily involve 2 distinct phases: accessing space and in-space transfer. The current approach predominantly employs a single multistage rocket to accomplish both phases in a single operation. However, this integrated model significantly constrains payload capacity for medium-high earth orbits and deep-space missions.

In this study, we propose a decoupled design for space access and in-space transfer. This design introduces a novel in-space transfer transportation system enabled by cryogenic propellant refueling in orbit. The proposed approach has the potential to substantially enhance payload capacity for medium-high earth orbits and deep space, while simultaneously reducing transportation costs.

## Analysis of the Current Space Transportation Model

Increasing the launch scale of rockets is the most direct and effective way to enhance payload capacity. The development of heavy-lift launch vehicles has become a dominant trend, exemplified by the rapid advancement of reusable high-capacity rockets such as the “Super-Heavy Starship” system. Currently, the payload capacity of low-earth-orbit launch vehicles has reached the 100-ton class, with the Super-Heavy Starship achieving a maximum launch mass of 5,000 to 10,000 tons [16]. However, further significant increases in rocket scale face considerable challenges due to limitations in engine performance, structural materials, manufacturing techniques, and spatial configuration. Additionally, the adoption of reusable technologies introduces design trade-offs, including aerodynamic thermal protection for reentry, landing mechanisms, and retro-propulsion systems, which reduce payload capacity. Consequently, achieving substantial further improvements in the size and capacity of single rockets has become extremely difficult.

Current space transportation models predominantly rely on single multistage rockets to perform both space access and in-space transfer within a single operation. In this approach, the rocket’s final stage and payload are accelerated by the booster stages to the required velocity and altitude. The final stage then delivers the payload to its target orbit. This design principle is rooted in the Tsiolkovsky multistage rocket equation. However, for medium-high earth orbit and deep-space missions, payload capacity is directly constrained by the size of the rocket’s final stage, which itself is limited by the overall rocket scale. Since further increases in rocket scale are challenging, the payload capacity for these missions is severely restricted. For instance, a heavy-lift rocket (lift-off mass 5,000 t) may only achieve a payload capacity of approximately 1.0% of its launch mass (50 tons) to a trans-lunar injection orbit, with a payload capacity of merely 0.1% of its launch mass (5.0 tons) for a round-trip lunar mission. Furthermore, the dual-phase requirements of space access and in-space transfer impose conflicting environmental and design constraints on single-rocket systems, further limiting their payload capacity. During the space access phase, rockets must endure extreme aerodynamic and thermal loads as well as Earth’s deep gravitational well. These conditions necessitate aerodynamic shaping, thermal protection systems, and strict thrust-to-weight ratio designs. In contrast, the in-space transfer phase operates in a microgravity environment with no aerodynamic or gravitational well constraints, eliminating the need for aerodynamic design and thermal protection. This phase instead requires optimization for long-term exposure to microgravity, thermal radiation, and electromagnetic radiation. Table 1 summarizes the contrasting environmental and design requirements for these 2 phases.

**Table 1.** Comparison of flight environments and design constraints between space access and in-space transfer

Phase	Space access	In-space transfer
Atmospheric environment	Experiences severe aerodynamic and thermal loads (aerodynamic forces of several tons to hundreds of tons, aerodynamic heating up to 10^6^ W/m^2^); requires aerodynamic shaping and thermal protection	No aerodynamic loads; aerodynamic shape constraints are unnecessary
Gravitational environment	Must quickly overcome Earth’s gravity during lift-off and ascent; thrust-to-weight ratio must exceed 1; maximum g-force can exceed 5, requiring high structural load capacity; low fault tolerance during ascent	Operates in a microgravity environment (<10^−3^g); thrust-to-weight ratio can be much lower; structural load requirements are minimal
Space thermal radiation	Minimal impact	Requires consideration of long-term cryogenic propellant boil-off control under thermal radiation conditions
Space particle radiation	Minimal impact	Requires shielding and protection against prolonged exposure to particle radiation in space

Future large-scale space missions, such as cislunar exploration and development, manned Mars missions, the construction of extensive space infrastructure, asteroid exploration and exploitation, and deep-space exploration, demand significantly enhanced in-space transfer capabilities [11–13]. For example, a single manned Mars mission requires a crew spacecraft mass of 65 tons for Earth-to-Mars transfer, while cargo spacecraft may exceed 200 tons [12], far surpassing the capacity limits of current launch vehicles. Moreover, the substantial cost reductions necessary for future large-scale space exploration underscore the need for a fundamental shift in space transportation paradigms.

## A New Decoupled Model for Space Transportation and its Advantages

### A novel decoupled design for space access and in-space transfer

**Fig. 1.**
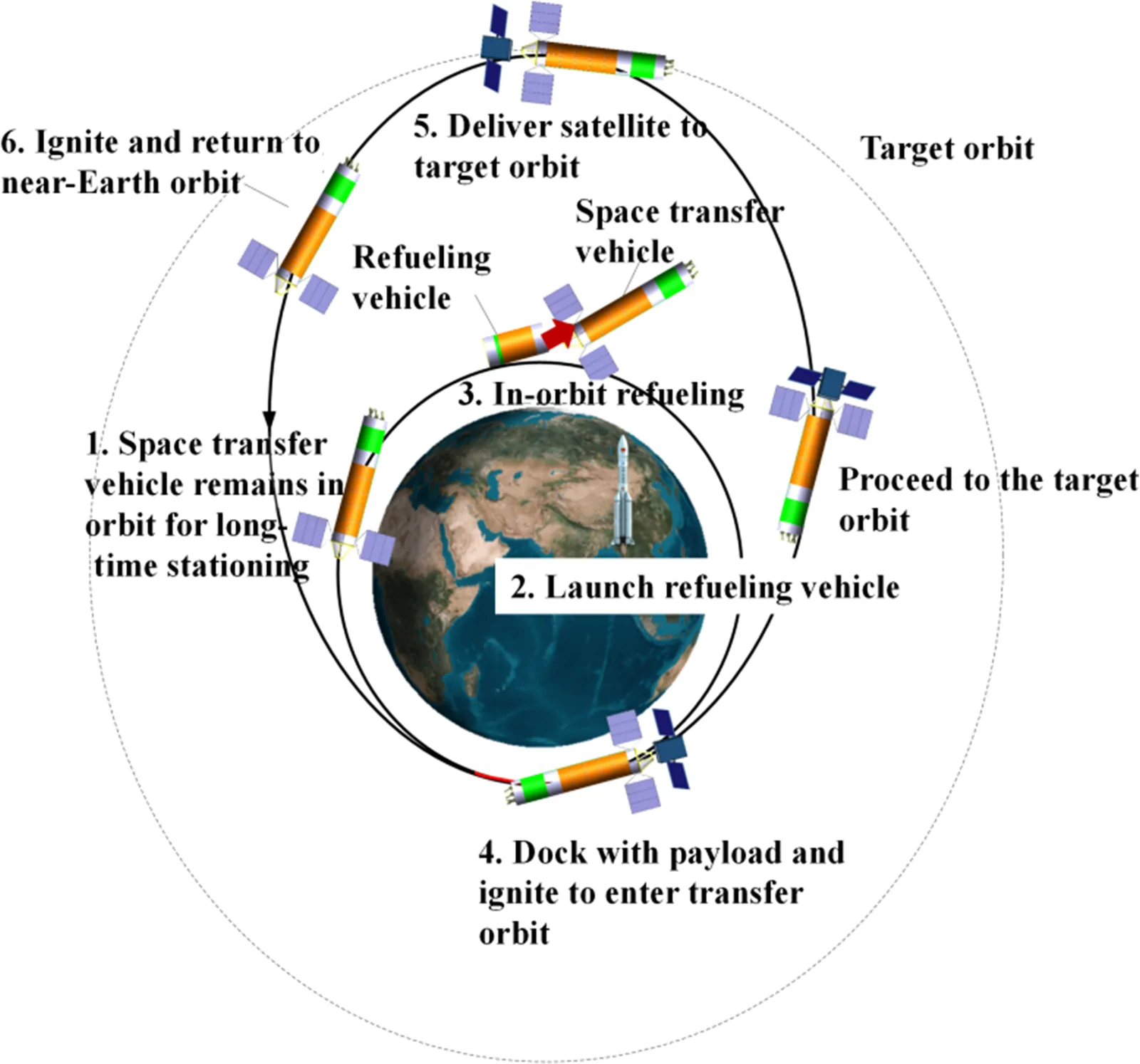
Schematic of decoupled Earth–space round trip/in-space transfer transportation based on in-orbit refueling.

This paper proposes a decoupled design for space access and in-space transfer. The core concept is to assign these 2 distinct phases of space transportation to separated systems: the launch vehicle and the in-space transfer vehicle. The launch vehicle is responsible for delivering payloads and propellants to the orbital (e.g., low-Earth orbit). In this orbit, a long-term, reusable in-space transfer vehicle is stationed. After docking with the payload and being refueled, the transfer vehicle delivers the payload to the target orbit. It then returns to its original station, awaiting refueling for subsequent missions. This process is illustrated in Fig. 1.

### Advantages of the new transportation model

The proposed decoupled transportation design overcomes the limitations of the current single multistage rocket approach. It significantly enhances transportation capabilities for medium-high earth orbits and deep space while substantially reducing transportation costs.

#### Transportation payload capability

On one hand, the maximum payload capacity of a launch vehicle can be utilized to deliver the dry mass (excluding propellant) of the in-space transfer vehicle to a parking orbit. The transfer vehicle can then be refueled in orbit, with the propellant mass typically being approximately 9 times the dry mass. This enables the transfer vehicle to achieve a scale more than an order of magnitude larger than the final stage of the launch vehicle. On the other hand, since the in-space transfer vehicle remains in orbit long term, it does not need to address the dual constraints of space access and in-space transfer environments. Instead, it can be independently optimized specifically for the conditions of orbital flight. For example, without considering other factors, and focusing only on ascent-phase loads, the structural load-bearing ratio of the Starship is 6.8 times greater than that of a long-term orbital transfer vehicle. The combination of increased scale and independent optimization allows the in-space transfer vehicle to significantly enhance its transportation capacity for higher orbits and deep space.

In terms of propellant selection for the in-space transfer vehicle, the Tsiolkovsky rocket equation indicates that high-specific-impulse propellants should be prioritized [17,18]. Cryogenic propellants, e.g., liquid hydrogen/liquid oxygen with a specific impulse of up to 460 s, or liquid oxygen/methane with a specific impulse of 380 s, offer higher efficiency and environmental benefits compared to conventional propellants such as nitrogen tetroxide/monomethylhydrazine (specific impulse ~300 s). For a given orbit transfer mission with a specific delta-v requirement, using cryogenic propellants instead of conventional ones can either drastically reduce the initial orbital departure mass or substantially increase payload capacity [19].

Even when considering the boil-off losses of cryogenic propellants during long-term operation in orbit, conventional propellants can achieve zero boil-off losses. However, when it comes to large-scale space transfer transportation, cryogenic space transfer vehicles are relatively large in size, and the boil-off rate of their cryogenic propellants is easier to control compared to that of smaller-scale ones. Here, liquid oxygen and methane can achieve a daily boil-off rate of 0% to 0.3%, and liquid hydrogen can achieve a daily boil-off rate of 0.1% to 1%. Table 2 provides a comparative analysis of the required propellant mass for a typical space transfer mission using various types of propellants while considering the boil-off losses of cryogenic propellants.

**Table 2.** Comparison of propellant mass requirements for on-orbit refueling in typical space transportation missions (assuming a 10-ton payload)

Mission	Delta-v requirement (m/s)	Propellant type	Specific impulse (s)	Required propellant mass (tons)
LEO to MEO round trip	7,250	Conventional (N_2_O_4_/UDMH)	316	697
		Liquid oxygen/methane	380	148–151
		Liquid hydrogen/oxygen	460	72–74

LEO, low earth orbit; MEO, medium earth orbit; UDMH, unsymmetrical dimethyl hydrazine

Taking China’s most capable launch vehicle, the Long March-5, as an example, its capacity can be used to deliver the dry mass of a novel in-space transfer vehicle into a low-Earth orbit. After on-orbit refueling, the system’s orbital departure mass can increase from 24 tons to approximately 240 tons, representing a significant enhancement in its transportation capability. As shown in Table 3, the introduction of on-orbit refueling also enables the in-space transfer system to be reused multiple times, significantly reducing overall transportation costs.

**Table 3.** Comparison of payload capacity between multistage rocket one-time arrival model and in-orbit refueling transportation model

Transportation model	Scale of departure of the transportation system in orbit (tons)	Carrying capacity (tons)			
		LTO	LMO	Round trip between LEO and GEO	Round trip between Earth and Moon
Multistage rocket one-time arrival model	24	8.3	5.5	0.2	0.34
New transport model	240	185	144	19	21

LTO, lunar transfer orbit; LMO, low Mars orbit; GEO, geostationary earth orbit

It is worth noting that SpaceX’s “Super-Heavy Starship” system also plans to use on-orbit refueling to enhance deep-space transportation capabilities. Because the overall design of the Super-Heavy Starship system remains that of a 2-stage rocket, which is not the optimal configuration for deep-space transportation, on-orbit refueling is required to enhance its capabilities for deep-space missions. Since the Super-Heavy Starship is still a 2-stage rocket, its design still integrates both the space access and in-space transfer phases. As a result, the Starship second stage must feature an aerodynamic shape, along with aerodynamic thermal protection, canard wings, and landing mechanisms. Additionally, its propulsion system incorporates 2 types of engines: vacuum-optimized Raptor engines for the ascent phase and sea-level Raptor engines for the landing phase, each operating during different mission stages. Furthermore, the system must address challenges such as controlling cryogenic propellant boil-off during on-orbit storage. These design considerations, which aim to accommodate both phases, inevitably reduce the efficiency of Starship compared to systems optimized for a single phase of transportation.

#### Transportation costs

The proposed decoupled transportation design significantly reduces costs for medium-high earth orbit and deep-space missions. On one hand, the in-space transfer vehicle remains in orbit long term, enabling on-orbit refueling and reuse. When combined with reusable Earth-to-orbit vehicles, this approach establishes a fully reusable transportation system spanning from Earth to deep space, resulting in substantial cost reductions. On the other hand, the Earth-to-orbit and in-space transfer vehicles can be independently optimized for their respective flight environments without being constrained by dual-environment requirements. This optimization maximizes the reusability and operational lifespan of the vehicles, further reducing overall costs.

### Comparative analysis of propulsion technologies for in-space transfer

Under the proposed decoupled transportation model, the in-space transfer phase discussed earlier primarily utilizes chemical propulsion. Beyond chemical propulsion, alternative technologies such as electric and nuclear propulsion are also the options. As shown in Table 4, a comparative analysis of these propulsion technologies highlights that chemical propulsion remains the most practical and feasible choice for large-scale in-space transportation at present.

**Table 4.** Comparative analysis of propulsion technologies for in-space transfer

Performance	Chemical propulsion	Electric propulsion	Nuclear thermal propulsion	Nuclear electric propulsion
Safety and usability	High; suitable for Earth and low-orbit applications	High; suitable for Earth and low-orbit applications	Low; safety concerns due to inability to return nuclear devices to low orbit	
Thrust	Hundreds of tons	Sub-newton levels	Hundreds of tons	Up to several newtons
Transfer time (e.g., Earth–Moon)	Days	Years	Days	Years
Specific impulse (s)	460	4,000	800	4,000
Structural dead weight ratio	~10%	~30%	~40%	~85%
Technology readiness	7–9	6–7	3	3–4
Service life	Long	Limited by radiation effects on solar panels during the process of high- and low-orbit transportation	TBD	TBD

TBD, to be determined

## Design Challenges

### Optimization of the space transportation system

Future large-scale space exploration and development will require extensive space access and in-space transportation capabilities [14,18]. Under the proposed decoupled transportation model, Earth-to-orbit vehicles and in-space transfer vehicles will work together to accomplish these missions, necessitating comprehensive optimization of the entire space transportation system. First, the design of the Earth-to-orbit vehicles and in-space transfer vehicles must be optimized to meet mission requirements. Their configurations, including scale, capacity, and quantity, need to be holistically designed and balanced to avoid excessive complexity. Second, future large-scale and multitype space missions, including Earth-orbit operations (low and high), cislunar transport, asteroid exploration, planetary missions (e.g., Mars), solar exploration, and outer solar system missions, will involve numerous interconnected elements. These include ground launch sites, Earth-to-orbit vehicles, in-space transfer vehicles, refueling vehicles, satellites, payloads, transfer orbits, and more. The system must also account for a wide range of design parameters, such as vehicle types, sizes, quantities, refueling orbits, and rendezvous schedules. Moreover, the spatiotemporal relationships between orbital vehicles and celestial bodies are constantly evolving, resulting in a system with high dimensionality, strong interdependencies among elements, and sensitivity to spatiotemporal constraints. These factors make the comprehensive optimization of future space transportation systems a significant challenge.

### Large-scale and efficient Earth-to-orbit transportation

#### Reusable structural design under harsh thermo-mechanical environments

Earth-to-orbit vehicles face significant challenges in enduring repeated entries into space, particularly during multiple high-speed orbital reentries. These challenges include harsh alternating thermo-mechanical environments, stringent requirements for rapid inspection and maintenance, and the need for high-performance reusability. During high-speed reentry, aerodynamic heating can reach heat flux densities in the megawatt-per-square-meter range, with high total heat loads. Additionally, the extreme and complex flight environments, nonlinear interactions of alternating thermo-mechanical loads on complex structures, and the limited availability of design and test samples exacerbate these difficulties. Traditional ablative thermal protection systems are inadequate to meet the high demands for rapid maintenance and reliable reuse. Therefore, advanced aerothermodynamic-structural prediction methods must be developed to accurately model the harsh thermo-mechanical environments and uncover the underlying mechanisms of multifield coupling, enabling the design of novel load-bearing and thermal protection integrated structures. Fundamental research on fatigue and damage mechanisms under high-frequency alternating stress and rapid heating/cooling conditions is essential to support rapid inspection and maintenance. Additionally, thermal protection designs combining active and passive approaches should be pursued, leveraging energy transport-dissipation matching principles to achieve comprehensive thermal management and meet the high reliability requirements for reusable systems.

#### Complex environment perception and intelligent morphing flight for ultra-high-speed flight

To meet the future demand for routine space access flights, the number of space access missions is increasing. On the one hand, the vehicle experiences a large span of airspace and a complex thermal environment, which makes it difficult for a single-configuration vehicle layout program to accurately match the environmental requirements of each flight phase. It is necessary to construct real-time sensing and identification of flight environment, online intelligent solution of non-constant flow field, coupled simulation method of vehicle motion, and flight control integration; develop numerical simulation technology of vehicle aerodynamic/control integration flight; research on adaptive intelligent variant configuration technology that can sense the flight environment in real time; and realize the adaptive intelligent variant configuration flight that accurately matches the various flight phases. On the other hand, with the increasing number of low-orbit giant constellations, space debris, and aerial vehicles, the orbits and surrounding areas for future large-scale round-trip transportation between the earth and the heavens have formed an extremely complex and dense space environment. This staggered distribution of space targets makes the carrier face changing mission environments and high collision risks during the round trip between heaven and earth, which makes the traditional ground-dependent control mode unable to meet the flight requirements. Therefore, it is necessary to study how to realize online fast and accurate sensing of the flight situation of the carrier and dynamic risk assessment of the dense environment through multi-sensor information fusion, to realize flight decision-making and mission replanting under limited on-board computational resources based on flight situational awareness and risk assessment, and to realize optimal avoidance guidance under dynamic strong process constraints with comprehensive consideration of the dynamic constraints of the dense environment and the constraints of the drop zone.

#### High-precision and high-safety landing

Earth-to-orbit vehicles are expected to feature full reusability, rapid launch capability, and multiple reuse cycles. This introduces challenges such as high-safety recovery of rocket stages near the original launch site. The reentry process for rocket stages involves complex characteristics, including transitions across multiple atmospheric and space domains, wide velocity ranges, uncertainties in environmental and dynamic model parameters, significant aeroelastic effects, and low vibration frequencies. These factors make high-precision and high-safety landing guidance and control particularly challenging. First, the flight profile for reentry of large-mass vehicles is highly complex and subject to stringent constraints, including dynamic pressure, overload, and heat flux density. The process involves prolonged unpowered reentry and multiple engine restarts, with reentry point deviations and various internal and external disturbances severely affecting guidance accuracy. Traditional open-loop or tracking guidance methods cannot meet the sub-meter-level precision landing requirements. Second, the reentry process spans multiple flight phases, covering distinct atmospheric conditions from high altitudes to ground level. The operational characteristics of actuators vary significantly across these phases, necessitating the design of control methods tailored to the environmental and actuator characteristics of each phase. Third, under the influence of aerodynamic-thermal-structural coupling, large-mass vehicles exhibit pronounced time-varying elastic modal characteristics. The discrepancies between predefined aerodynamic parameters and actual flight conditions further amplify these effects, increasing the likelihood of actuator saturation.

#### Autonomous health management and capability assessment

Large-scale, efficient Earth-to-orbit vehicles are characterized by high flight frequencies, stringent reliability requirements, and extended operational lifespans. This necessitates the development of autonomous health management and capability assessment systems to enable comprehensive monitoring of the vehicle’s health and performance. However, achieving these objectives poses several challenges. First, high-precision sensor arrays must be integrated to capture environmental variations and key parameters of the vehicle’s structural, electrical, and propulsion subsystems during flight. This supports online fault diagnosis and enhances autonomous fault recognition capabilities. Second, advanced intelligent technologies, including big data analytics and machine-learning algorithms, must be leveraged to deeply analyze sensor data and perform pattern recognition. This improves monitoring efficiency and enables early warning of potential failures, facilitating autonomous health management. Third, by synthesizing internal and external sensing information, the remaining capability of the transportation system must be evaluated in real time. Accurate prediction and assessment of capability boundaries are crucial for supporting reliable flight operations and providing critical information for decision-making and control.

#### Ground agile maintenance and support

Agile maintenance and support for large-scale, efficient Earth-to-orbit transportation missions are critical for ensuring safe and reliable space transportation. Maintenance and repair strategies must be adapted based on mission scenarios, system health status, and available support resources, enabling on-demand maintenance and repair operations. First, Earth-to-orbit vehicles are subjected to multiple cycles of high-magnitude alternating thermo-mechanical environments. Rapid and agile evaluation of the safety and reliability of structural components for subsequent launches is essential. However, these vehicles feature complex structures with numerous components, some of which are embedded internally, making inspection challenging without disassembly. The difficulty is compounded by the limited availability of inspection resources. Therefore, new non-destructive, agile, and accurate inspection techniques are needed, including the development of focal plane array detection systems capable of simultaneously evaluating the health status of large structural areas. Second, after completing structural health evaluations and fault localization, agile maintenance and repair strategies must be implemented. These strategies are diverse, and the available evaluation data samples are often limited. To address this, it is necessary to integrate multiple types of small-sample evaluation data within a short time frame, aiming to balance cost-effectiveness and reliability. Agile maintenance and repair logic decisions must then be rapidly executed to identify the optimal maintenance plan and efficiently implement repair strategies. These requirements present significant challenges in achieving timely and effective maintenance operations.

### In-space transfer transportation based on on-orbit refueling

The development of a novel in-space transfer transportation system based on on-orbit refueling offers transformative potential for enhancing transportation capabilities to medium-high earth orbits and deep space, presenting significant engineering and practical value. However, this new transportation model also introduces numerous technical challenges for in-space transfer operations.

#### Overall design of the novel in-space transfer vehicle

The overall design of the novel in-space transfer vehicle presents significant challenges due to its large scale, substantial cryogenic propellant refueling requirements, and long-term orbital operation. Its thermo-mechanical environment and design requirements differ significantly from those of traditional launch vehicles and satellites, necessitating advanced optimization techniques. Compared to the final stages of current cryogenic launch vehicles, the novel transfer vehicle is substantially larger (by several to tens of times) and operates in an orbital environment with reduced load conditions (several times lower). It is not constrained by aerodynamic or thermal requirements and benefits from lower thrust-to-weight ratios, reduced overload, and a more favorable mechanical environment during transfer missions. However, the vehicle is subjected to the complexities of long-term orbital exposure, requiring considerations such as cryogenic propellant boil-off control under the space thermal environment. Traditional design approaches for rocket upper stages are no longer applicable to the new transfer system based on on-orbit refueling. In addition to achieving high transportation efficiency, the transfer vehicle must support multiple refueling cycles, enable convenient in-orbit maintenance, and offer adaptability for various mission profiles. These requirements impose significant challenges on the vehicle’s overall structural configuration, including the design of large, ultra-thin cryogenic tanks and other key components, as shown in Table 5. Consequently, under these new environments, requirements, and constraints, the traditional design methodologies for cryogenic launch vehicles and their upper stages are inadequate. It is imperative to establish a new framework for the overall design and optimization of reusable in-space transfer vehicles tailored for on-orbit operations.

**Table 5.** Comparison of design constraints between Earth-to-orbit vehicles and in-space transfer vehicles

Performance	Earth-to-orbit vehicles	In-space transfer vehicles
Atmospheric environment	Severe aerodynamic and thermal loads	No aerodynamic or thermal constraints
Structural load	High structural loads	Low structural loads
Time in orbit	Short duration (≤1 h)	Long duration (months to years)
Refueling location	Ground-based refueling	On-orbit refueling
Space thermal/radiation environment	Minimal impact	Significant impact due to long-term exposure
Maintenance	Ground-based maintenance	In-orbit maintenance

#### Cryogenic propellant management in microgravity

Cryogenic propellant management in a microgravity environment presents significant challenges, including the long-term, low-loss storage of large quantities of cryogenic propellants and the efficient transfer of propellant at high flow rates. Cryogenic propellants, such as liquid hydrogen at 20 K, liquid oxygen at 90 K, and liquid methane at 112 K, require on-orbit storage for durations ranging from several months to years. The propellant tanks may have diameters of 5 m or more, with total propellant masses reaching hundreds of tons and transfer flow rates up to 10 m^3^/min. Additionally, long-term operations in orbit involve diverse energy requirements, such as power supply, attitude and orbit control, and pressurization systems. Key technical challenges include developing methods to suppress evaporation and maintain phase stability for cryogenic propellants in space, enabling long-term, low-loss storage of large quantities of cryogenic propellants; efficiently pre-cooling cryogenic transfer system pipelines and tanks, initially at higher temperatures, to the target temperature required for stable transfer, using minimal propellant and achieving this within a shorter timeframe; and addressing the random distribution of gas and liquid phases in microgravity conditions during propellant transfer. Solutions are needed to achieve stable gas–liquid phase separation and minimize pressure and flow-rate fluctuations during high-flow-rate cryogenic propellant transfer.

#### Design and adaptive control of on-orbit cryogenic refueling interfaces

The design and adaptive control of cryogenic refueling interfaces face challenges such as optimizing docking mechanisms for high adaptability across multiple modes, ensuring seal integrity under significant cryogenic deformation, and achieving flexible, autonomous control under multitask, multi-constraint conditions. Specific challenges include designing docking mechanisms capable of accommodating large mass ratio variations (up to an order of magnitude between the transfer vehicle and refueling vehicle) and significant positional misalignments under highly variable thermal environments. The docking system must exhibit strong adaptive buffering capabilities while satisfying multiple objectives, including workspace, load-bearing capacity, and flexibility; ensuring the sealing performance of refueling interfaces under the influence of cryogenic deformation and propellant transfer pulsations, with interfaces maintaining reliable sealing after completing multiple on-orbit refueling cycles; and developing adaptive control strategies for docking mechanisms during dynamic changes in the center of mass, autonomously adjusting control parameters based on the mass relationship between the transfer and refueling vehicles in various tasks, dissipating impact energy, and achieving flexible, adaptive docking and cryogenic propellant transfer under changing conditions.

#### Position and attitude control of the launch vehicle with on-orbit refueling massive real-time changes

There are many technical challenges in the aspect of vehicle attitude control with on-orbit high mass real-time changes, such as characterization of mechanical coupling effects in the process of mass rapid exchange, identification and evaluation of dynamic characteristics, attitude control for diverse refueling missions, and so on. Unlike conventional on-orbit injection of low-flow room-temperature propellants, on-orbit mass/diverse injection of large isomer cryogenic propellants presents the following 4 difficulties for control system design. First is how to establish the combined body dynamics model for the control system design under the coupling of the time-varying disturbance of gas/liquid 2-phase flow motion and the rigid/elastic/wobbling motion during the mass transfer of combined body cryogenic propellant in microgravity; second, how to use the limited information to carry out the online identification and estimation of the parameters of the mass properties of the combined body, the non-continuous slosh mass disturbance and the elastic characteristics under the influence of excitation and measurement constraints, complex disturbance, etc., and evaluate the identification accuracy and availability; third, how to achieve the trade-offs among multiple optimization objectives, such as orbit parameter deviation, propellant consumption, radiation, and attitude adjustment angle, and achieve high precision orbit keeping while eliminating the effects of small overloads; the fourth is how to solve the problems of strong adaptation and multiobjective optimal control of the multi-injection attitude of the combination under the influence of large range changes of body characteristics (the maximum difference of mass before and after injection is 10 times), multisource interference, and rigid/projectile/wobbling coupling, and achieve high-quality attitude control.

## Conclusion

The current space transportation model primarily relies on single multistage rockets to accomplish both space access and in-space transfer phases in a single mission. This paper proposes a novel 2-phase transportation model that decouples space access and in-space transfer.

• In the proposed model, Earth-to-orbit vehicles are responsible for space access, while in-space transfer vehicles handle orbital transfer. This separation allows for independent optimization of vehicle designs according to their specific flight environments. Additionally, on-orbit refueling enables long-term, reusable operations for in-space transfer vehicles. For large-scale space transportation, employing cryogenic chemical propulsion technology for in-space transfer vehicles is the most practical and feasible option.
• The new transportation model significantly enhances payload capacity for medium-high earth orbits and substantially reduces launch costs.
• The implementation of this model requires comprehensive optimization of the entire transportation system, including the refinement of vehicle configurations.
• Efficient Earth-to-orbit transportation faces several technical challenges, including reusable structural design under harsh thermo-mechanical environments, intelligent flight systems, and high-precision, high-safety landing.
• Long-term in-space transportation must address challenges related to vehicle design, cryogenic propellant management in the orbital environment, refueling interface design, and on-orbit refueling control.

## Data Availability

All data required to draw the conclusions of this study are available within the article. Additional data may be obtained from the corresponding author upon reasonable request. There are no restrictions on the availability of the data related to this work.
